# Achieving treatment goals in older breast cancer patients receiving neoadjuvant chemotherapy

**DOI:** 10.1038/s41598-025-93203-1

**Published:** 2025-03-21

**Authors:** Eda Caliskan Yildirim, Elif Atag, Huseyin Salih Semiz, Olcun Umit Unal, Mehmet Uzun, Suleyman Ozkan Aksoy, Merih Guray Durak, Aziz Karaoglu

**Affiliations:** 1 Department of Internal Medicine, Division of Medical Oncology, Dokuz Eylul University, Izmir, 35340 Turkey; 2 Department of Internal Medicine, Health Sciences University Izmir Faculty of Medicine, Izmir, Turkey; 3 Department of General Surgery, Dokuz Eylul University, Izmir, Turkey; 4 Department of Pathology, Dokuz Eylul University, Izmir, Turkey

**Keywords:** Breast cancer, Neoadjuvant chemotherapy, PCR, Older patients, Downstaging, Breast cancer, Surgical oncology, Chemotherapy

## Abstract

Neoadjuvant chemotherapy (NAC) is well-established for locally advanced breast cancer, even in the early stages, especially in HER2-positive and triple-negative cases. However, the effect of chronologic age on NAC response remains controversial. This study investigates the efficacy and outcomes of NAC in older patients with breast cancer, compared to a younger cohort, to address the current knowledge gap. 535 patients who received NAC followed by curative surgery from 2010 to 2021 were retrospectively analyzed. We evaluated breast and axillary downstaging, pathologic complete response (pCR), and post-treatment toxicities. Data were stratified by age, with patients aged 65 years and older representing the older group. Anthracycline-based chemotherapy was prevalent (97.6%) and favored younger patients who received a dose-dense anthracycline regimen (71.7% vs. 38.5%, *p* < 0.001). Surgical outcomes, breast and axillary downstaging, and the pathologic complete response showed no age-related differences. Grade 3–4 toxicity was higher in older patients (71% vs. 46.4%, *p* < 0.01). Older patients treated with NAC achieve comparable outcomes to younger patients, supporting personalized treatment. Chronologic age should not dictate treatment decisions, emphasizing the need for comprehensive evaluation for optimal geriatric patient care.

## Introduction

Breast cancer is a major global health problem and its incidence increases with age. Approximately 45% of all breast cancer cases are diagnosed in patients over the age of 65^1^. However, the likelihood of breast cancer mortality increases with advancing age at diagnosis^2^.

With advances in neoadjuvant chemotherapy (NAC), the approach to managing locally advanced breast cancer has evolved. Neoadjuvant treatment has gained prominence not only in locally advanced stages but also in early-stage human epidermal growth factor receptor 2 (HER2) positive and triple-negative breast cancer^3^. However, there is a notable lack of research on the use of neoadjuvant chemotherapy in older patients with breast cancer. This knowledge gap limits our current understanding of the associated benefits and risks, and underscores the need for careful case-by-case assessment^4^. Despite the increasing use of NAC in older patients, the effect of chronological age on response and outcome remains debated. Existing data suggest that NAC results in comparable rates of pathologic complete response (pCR) in older patients with HER2-positive breast cancer as compared to their younger counterparts. In addition, several patients showed improved prognosis even without achieving pCR^5^.

The decision to administer neoadjuvant chemotherapy in older patients requires careful consideration. Age-related factors, such as comorbidities, impaired organ function, and frailty, pose unique challenges in the elderly population^6^. Concerns about tolerability, toxicity, and potential overtreatment often influence clinical decisions, leading to undertreatment or avoidance of NAC altogether. However, withholding effective treatment based solely on age may compromise outcomes and limit the therapeutic options available to older patients^7,8^.

The primary objective of this study is to determine whether chronological age influences the efficacy of neoadjuvant chemotherapy in older patients with breast cancer. Specifically, we aim to assess the rates of breast downstaging and axillary downstaging, as well as pathologic complete response, in both age groups. In addition, we will compare the use of anthracycline-based chemotherapy regimens, surgical approaches (breast-conserving surgery and sentinel lymph node dissection), and post-treatment toxicities between the two cohorts.

## Results

A total of 535 patients were enrolled. 12% of the patients (*n* = 69) were aged 65 years and older, with a mean age of 71 ± 4 years. Comorbidities were assessed using the Charlson Comorbidity Index (CCI). The geriatric cohort was significantly more likely to have a CCI of 3 or higher than the younger cohort (82.6% vs. 5.8%, *p* = 0.000). Diabetes mellitus, hypertension and heart disease were significantly more common in elderly patients and are summarised in Table 1. There were no statistically significant differences in the clinical stage and pathologic characteristics between patients aged 65 years and older and those younger than 65 years (*p* > 0.05). Regarding clinical subtypes, HR-positive HER-2 negative breast cancer comprised approximately half of the cases in both age groups. The rate of triple-negative breast cancer was higher in the group aged 65 years and older, while the rate of HER2-positive breast cancer was higher in the group younger than 65 years. However, these differences did not reach statistical significance (Table 1).

**Table 1:** Clinicopathologic characteristics.

Clinicopathologic characteristics	All cohort (*n* = 535)	< 65years (*n* = 466)	≥ 65 years (*n* = 69)	*p* value
Age (mean ± SD)	51 ± 11	48 ± 9	71 ± 4	**< 0.001**
Charlson Comordity Index(CCI) (n(%)) 0 1 2 ≥ 3	224(41.9) 133(24.9) 94(17.6) 84(15.7)	224(48.1) 133(28.5) 82(17.6) 27(5.8)	0 0 12(17.4) 57(82.6)	**< 0.000**
ECOG-PS(n(%)) 0 1 2	485(91.2) 46(8.6) 1(0.2)	444(95.9) 19(4.1) 0	41(59.4) 27(39.1) 1(1.4)	**< 0.000**
Diabetes Mellitus(n(%)) Yes No	80(15) 455(85)	57(12.2) 409(87.8)	23(33.3) 46(66.7)	**< 0.000**
Hypertension(n(%)) Yes No	125(23.4) 210(76.6)	81(17.4) 385(82.6)	44(63.8) 25(36.2)	**< 0.000**
Heart disease(n(%)) Yes No	23(4.3) 512(95.7)	13(2.8) 453(97.2)	10(14.5) 59(85.5)	**< 0.000**
Clinical T stage(n(%)) cT1 cT2 cT3 cT4	69(13.1) 275(52.2) 103(19.5) 80(15.2)	59(12.9) 244(53.2) 90(19.6) 66(14.4)	10(14.7) 31(45.6) 13(19.1) 14(20.6)	0.509
Clinical N stage (n(%)) cN0 cN1 cN2 cN3	47(8.9) 233(43.9) 223(42) 28(5.3)	39(8.4) 209(45.2) 190(41.1) 24(5.2)	8(11.6) 24(34.8) 33(47.8) 4(5.8)	0.415
Tumor grade(n(%)) 1 2 3	21(4) 273(51.8) 233(44.2)	15(3.3) 241(52.5) 203(44.2)	6(8.8) 32(47.1) 30(44.1)	0.085
Ki67(n(%)) <%30 >%30	179(34.7) 337(65.3)	154(34.1) 297(65.9)	25(38.5) 40(61.5)	0.494
Clinical subtypes(n(%)) Luminal A Luminal B- HER2 negative HER2 positive Triple negative	44(8.2) 225(42.1) 169(21.7) 96(18)	35(7.5) 197(42.4) 153(32.9) 80(17.2)	9(13) 28(40.6) 16(23.2) 16(23.2)	0.059
ER status(n(%)) Positive Negative	379(71) 155(29)	335(72) 130(28)	44(63.8) 25(36.2)	0.158
PR status(n(%)) Positive Negative	297(55.6) 237(44.4)	265(57) 200(43)	32(46.4) 37(53.6)	0.098

SD: standard deviation, HER2: human epidermal growth factor receptor, ER: estrogen receptor, PR: progesterone receptor.

All patients received a taxane-containing regimen ( paclitaxel or docetaxel) and 97.6% received anthracycline-based chemotherapy. A total of 13 patients did not receive anthracycline-based treatment, and 4 of them (30.7%) were 65 years of age or older. The use of dose-dense AC (ddAC) was 71.7% in the group younger than 65 years compared to 38.5% in the group 65 years and older (*p* < 0.001). Breast-conserving surgery was performed in 38% of the younger group and 31.9% of the older group (*p* > 0.05). The overall rate of sentinel lymph node dissection was approximately 39% of the entire population, and there was no statistically significant difference between the age groups (Table 2).

**Table 2:** Treatment-related characteristics of patients.

Treatment-related characteristic	Overall Population (*n* = 535)	< 65 years (*n* = 466)	≥ 65 years (*n* = 69)	*p* value
Anthracycline use(n(%))* ddAC/EC AC/EC/FAC/FEC	169(32.4) 352(67.6)	129(28.3) 327(71.7)	25(38.5) 40(61.5)	**< 0.00**
Surgery type(n(%)) BCS Mastectomy Bilateral mastectomy	199(37.2) 311(58.3) 24(4.5)	177(38) 268(57.5) 21(4.5)	22(31.9) 44(63.8) 3(4.3)	0.328
Lymph node (n(%)) SLNB ALND	207(38.6) 328(61.4)	181(39) 285(61)	25(36.2) 44(63.8)	0.649
pCR( n(%))	147(27.5)	124(26.6)	23(33.3)	0.24
Residual cancer burden pCR-RCBI RCBII-RCBIII	213(39.9) 321(60.1)	185(39.8) 280(60.2)	28(40.6) 41(59.4)	0.90
Breast downstaging	424(81.1)	374(82.2)	50(73.5)	0.09
Axilla downstaging	338(64.1)	290(63.2)	40(70.6)	0.23

dd: dose-dense A: Adriamycin C: cyclophosphamide, E: epirubicin F: 5-fluorouracil BCS: breast-conserving surgery, SLNB: sentinel lymph node biopsy, ALND: axillary lymph node dissection, pCR: pathologic complete response, RCB: residual cancer burden.

*all patients received taxane( docetaxel or paclitaxel),13 patients( %2.4) did not received anthracycline.

When comparing the groups in terms of pathologic complete response, 26.6% was observed in patients younger than 65 years, while 33.3% was observed in patients aged 65 years and older (*p* = 0.24). A good response to chemotherapy, defined as the achievement of pathologic complete response or residual cancer burden -I (RCB) was observed in approximately 40% of both the younger and older age groups (Table 2).

Patient outcomes were examined separately based on clinical breast cancer subtypes. When evaluating treatment responses in HER2-positive breast cancer patients, the pathologic complete response (pCR) rate was 81.3% (*n* = 13) in patients aged 65 years and older, compared to 47.4% (*n* = 73)in patients younger than 65 years(*p* = 0.016). In triple-negative breast cancer patients, the pCR rate was 31.3% (*n* = 5) in the older group and 43.8% (*p* = 0.415)in the younger group. In HR + HER-2 negative breast cancer patients, the pCR rate was 13.5% (*n* = 5) in patients aged 65 years and older and 6.9% (*n* = 16) in patients younger than 65 years(*p* = 0.166). In multivariate logistic regression analysis, HER2 status, Ki67 > 30%, PR negativity, and high grade were identified as independent variables influencing pCR (Table 3).

**Table 3:** Predictive factors for pCR in univariate and multivariate analysis.

	Univariate Analysis HR (%95CI)	*p*-value	Multivariate Analysis HR (%95 CI)	*p*-value
Age > 65y vs. <65y	1.37(0.80–2.36)	0.24		
HER2-positive vs. HER2-negative	5.10(3.39–7.66)	**0.000**	3.86(2.45–6.08)	**0.000**
Ki67>%30 vs. Ki67<%30	3.43(2.11–5.57)	**0.000**	2.02(1.14–3.57)	**0.015**
Grade 3 vs. Grade 1–2	3.91(2.60–5.88)	**0.000**	1.93(1.17–3.19)	**0.009**
ER positive vs. ER negative	0.20(0.13–0.30)	**0.000**	0.54(0.29 − 1.00)	0.050
PR positive vs. PR negative	0.20(0.13–0.31)	**0.000**	0.47(0.26–0.87)	**0.016**
ddAC vs. AC	1.50(0.97–2.32)	0.064		

pCR: pathologic complete response, ER: estrogen receptor, PR: progesterone receptor, dd: dose-dense A: Adriamycin C: cyclophosphamide.

In logistic regression analysis, cT1-2, cN 0–1, and grade 3 were identified as independent predictors in assessing patients’ eligibility for breast-conserving surgery (Table 4). Meanwhile, HER2-positivity, Ki67 > 30%, PR negativity, cN0-1, and receiving ddAC were independent predictors when evaluating the factors that determine whether axillary lymph node dissection is necessary (Table 5).

**Table 4:** Predictive factors for breast-conserving surgery in univariate and multivariate analysis.

	Univariate Analysis HR (%95CI)	*p*-value	Multivariate Analysis HR (%95 CI)	*p*-value
Age < 65y vs. > 65y	1.30(0.76–2.24)	0.329		
HER2-positive vs. HER2-negative	1.23(0.85–1.79)	0.268		
Ki67>%30 vs. Ki67<%30	1.07(0.73–1.56)	0.714		
Grade 3 vs. Grade 1–2	1.62(1.14–2.32)	**0.007**	1.64(1.12–2.41)	**0.011**
ER negative vs. positive	1.07(0.72-0.1.57)	0.728		
PR negative vs. positive	0.94(0.66–1.34)	0.762		
cT1-2 vs. cT3-4	4.41(2.84–6.86)	**0.000**	4.15(2.65–6.50)	**0.000**
cN0-1 vs. cN2-3	1.93(1.35–2.77)	**0.000**	2.07(1.40–3.05)	**0.000**
ddAC vs. AC	1.36(0.93–2.01)	0.111		

ER: estrogen receptor, PR: progesterone receptor, dd: dose-dense A: Adriamycin C: cyclophosphamide.

**Table 5:** Predictive factors for avoiding axillary lymph node dissection in univariate and multivariate analysis.

	Univariate Analysis HR (%95CI)	*p*-value	Multivariate Analysis HR (%95 CI)	*p*-value
Age < 65y vs. > 65y	0.88 (0.52–1.49)	0.653		
HER2-positive vs. HER2 negative	1.92 (1.32–2.78)	**0.001**	1.73(1.13–2.64)	**0.011**
Ki67>%30 vs. Ki67<%30	1.57 (1.07–2.30)	**0.019**	1.46(0.95–2.24)	0.082
Grade 3 vs. Grade 1–2	1.31 (0.92–1.86)	0.133		
ER negative vs. ER positive	1.51(1.03–2.20)	**0.033**	1.3(0.73 − 2.30)	0.363
PR negative vs. PR positive	1.72(1.21–2.45)	**0.002**	1.76(1.04–2.99)	**0.033**
cT1-2 vs. cT3-4	1.40(0.96–2.04)	0.073		
cN0-1 vs. cN2-3	2.09(1.46–2.99)	**0.000**	2.26(1.51–3.37)	**0.000**
ddAC vs. AC	2.93 (1.93–4.46)	**0.000**	2.91 (1.86–4.57)	**0.000**

ER: estrogen receptor, PR: progesterone receptor, dd: dose-dense A: Adriamycin C: cyclophosphamide.

A total of 233 patients were clinically staged as N1, of which only 10% (*n* = 24) were older patients. pCR rates in cN1 patients were 22.5% (*n* = 47) in younger patients and 33.3% (*n* = 8) in older patients. Although the axillary downstaging rate favored older patients (78% vs. 50%, *p* = 0.01), only 37.5% of patients did not undergo axillary dissection ( 45% in the younger group, *p* = 0.48). Despite similar tumor downstaging rates, the rate of breast-conserving surgery is 29.2% in the older group compared to 47.8% in the younger group.

When all toxicities were considered together, a statistically significant higher rate of grade 3–4 toxicities was observed in older patients (71% vs. 46.4%, *p* < 0.001). Retrospective data limitations prevented the inclusion of Grade 1–2 toxicities, as these were not routinely recorded in the patient records. The most common Grade 3–4 toxicity in both age groups was myelosuppression, with neutropenia being the most frequently encountered issue. Neutropenia occurred in 24.6%of older patients and 14.6% of younger patients and was managed with granulocyte colony-stimulating factor (G-CSF). Following myelosuppression, the most common severe toxicity was peripheral neuropathy, which was reported in 6.4% of the young and 20.3% of the elderly. These common toxicities are followed by nausea and vomiting, skin toxicity and diarrhoea. In the entire cohort, hepatoxicity was developed in 6 patients, thrombotic events in 5 patients and cardiotoxicity in 3 patients. There were no treatment-related deaths.

Recurrence occurred in 84 patients (17%). In the group under 65 years of age, 17% of patients(*n* = 77) experienced a relapse while in the counterpart, 10.4% of patients developed recurrence (*p* = 0.176).

## Discussion

This study provides compelling evidence that breast cancer patients over the age of 65 achieve similar benefits from neoadjuvant chemotherapy (NACT) as their younger counterparts. Our findings challenge the common clinician biases against administering aggressive treatment regimens to older patients solely based on age. By demonstrating comparable rates of pathologic complete response (pCR), axillary downstaging, and surgical outcomes, this study reinforces the notion that age alone should not preclude the use of NAC.

Only 12% of our study population comprised older patients. Considering that approximately half of the women with breast cancer are over 65 years of age and that this proportion is increasing in favor of older patients over the years^9^, the low percentage of older patients in our study is noteworthy. The most likely reason for this is the avoidance of neoadjuvant chemotherapy (NACT) due to the presence of comorbid diseases and concerns about toxicity The higher proportion of triple-negative breast cancer in older patients may reflect selection bias due to the retrospective design of the study, as only patients deemed fit for neoadjuvant chemotherapy (NAC) were included. This highlights the importance of considering the inherent limitations of retrospective analyses when interpreting these results in older patients.

Selecting fit geriatric patients for NAC is a reasonable approach since treatment de-escalation is not commonly practiced in NAC protocols, and sequential anthracycline and taxane-based regimens are applied, which can be toxic for frail elderly patients. The European Society of Breast Cancer Specialists (EUSOMA) and the International Society of Geriatric Oncology (SIOG) recommend sequential regimens only for high-risk, fit elderly patients due to insufficient evidence in the general older population^10^. The underrepresentation of this population has historically limited the generalizability of clinical trial results and perpetuated uncertainties about treatment efficacy and safety in older adults. Our study adds to the growing body of evidence that older patients can tolerate and benefit from standard-of-care therapies when appropriately selected.

Meta-analyses have shown that dose-dense regimens provide an advantage in disease-free survival (DFS) and overall survival (OS) compared to standard regimens^11,12^. However, dose-dense regimens are not recommended for patients older than 70 years due to increased toxicity and insufficient efficacy. In our study, 38.5% of geriatric patients and 71% of younger patients received ddAC. The high rate of preference for ddAC regimens observed in our study may be due to the age limit for geriatric patients being set at 65 years and the selection of patients taking into account comorbidities. Furthermore, this study identified the use of ddAC regimens as an independent risk factor for avoiding axillary dissection. Especially in older patients, the prevention of lymphedema is very important for quality of life and is often the main indication for the use of NAC in clinical practice. One of the key clinical implications of these findings is the importance of personalized treatment planning for older patients. Geriatric assessments should be integrated into routine oncology practice to evaluate comorbidities, functional status, and frailty, enabling clinicians to stratify patients based on their fitness for treatment. Tailored approaches, such as the judicious use of dose-dense regimens in select older patients with good performance status, can maximize therapeutic benefits while minimizing toxicities.

The use of NAC is an effective strategy to avoid mastectomy and axillary dissection in locally advanced breast cancer. These surgical goals are at least as important in older patients as in younger ones. Opting for breast-conserving surgery (BCS) or sentinel lymph node biopsy (SLNB) instead of axillary dissection (AD) not only preserves the perception of body integrity and provides quality of life benefits in older patients but also reduces surgical morbidity and postoperative hospital stay^13,14^. When comparing two groups with similar clinical T and N stages and molecular subtypes, we found that the rates of axillary downstaging and breast downstaging were similar, resulting in comparable rates of BCS application and avoidance of AD. A subgroup analysis of cN1 patients, who had the highest likelihood of avoiding axillary dissection after NAC, revealed higher axillary downstaging rates in older patients. In this group, about 30% avoided AD and underwent SLNB instead.

There are a limited number of studies in the literature that examine the surgical outcomes of NAC concerning chronologic age. In a study by Williams et al., 651 patients with clinical T1-3N0-1 were analyzed, of which 75 patients were over 70 age. In this study, the conversion rate to BCS with NAC was 72% in patients ≥ 70 years of age, 74% in the 50–69 age group, and the rate of avoidance AD was 43% vs. 42%, respectively, with similar outcomes between the groups. Although older patients had a lower treatment completion rate, they still achieved these results^15^. In the study by Francys et al., 1383 patients with stage 1–3 breast cancer receiving NAC were divided into three age groups (≤ 40, 41–60, and ≥ 61 years). While conversion rates to BCS were similar across groups, among cN1 patients (*n* = 813), 52% of women ≤ 40 years of age avoided axillary lymph node dissection (ALND) with NAC, compared with 39% and 37% in the older groups (*p* < 0.001)^16^. Although studies are scarce, the general results suggest that older patients benefit from treatments as much as younger patients and our study results support this data trend.

In our study, the rates of pathologic response after NAC were similar between the older and younger groups (33.3% vs. 26.6%). The pCR rate in HER-2 positive disease was significantly higher in the older group compared to the younger group (81.3% vs. 47.4%). The higher treatment response in HER2-positive older patients compared to younger patients might be influenced by the imbalanced distribution of accompanying hormone receptor positivity within this subgroup(12.5% vs. 41.2%). These observations underscore the need for prospective studies to validate these findings and better understand the interplay of age, tumor biology, and treatment response.

Recurrence rates were also found to be similar between the two groups. Independent risk factors for pCR included ER and PR negativity, HER-2 positivity, grade 3 disease, and a Ki-67 greater than 30%. These results may be useful for patients facing the dilemma of whether to receive NAC. We face a serious dilemma, especially in older patients with HR+, HER-2 negative clinical N1 disease, because according to the RxPONDER study, if this group (RS ≤ 25) is considered low risk, endocrine therapy may be sufficient. In situations where we have to weigh the chance of avoiding AD with NAC against the patient’s chance of not receiving any chemotherapy, these factors may be helpful.

The higher toxicity rates observed in older patients, particularly Grade 3–4 toxicities such as myelosuppression and peripheral neuropathy, may reflect age-related changes in pharmacokinetics and pharmacodynamics, as well as the cumulative impact of comorbidities and reduced organ reserve. Despite these toxicities, the comparable treatment outcomes between age groups underscore the importance of patient selection and supportive care. These findings highlight the critical role of comprehensive geriatric assessment (CGA) in identifying patients who can tolerate intensive treatment and tailoring supportive care strategies to mitigate toxicities.

The strengths of our study are that treatment plans were determined by tumor boards at two large tertiary cancer centers and that separate analyses were performed for outcomes targeted with NAC. This collaborative approach underscores the value of interdisciplinary care in optimizing outcomes for older patients. Furthermore, systemic barriers, such as limited access to geriatrician and institutional biases toward less aggressive treatments, must be addressed. Initiatives to train clinicians in geriatric assessment and to develop age-specific treatment protocols are critical steps forward.

However, the small proportion of older patients (12%) in our study reflects a limitation that may reduce the statistical power for detecting significant differences in outcomes between the age groups. This limitation could lead to an underestimation of the variability within the older population and affect the generalizability of our findings to the broader elderly population. Future studies with larger cohorts of older patients are needed to confirm these results and strengthen the reliability of the conclusions. On the other hand, the inclusion of patients who could complete at least 80% of the planned neoadjuvant chemotherapy protocol constitutes an important selection bias. We chose such an inclusion criterion to minimize treatment heterogeneity, as local therapeutic approaches may differ between patients who were able to receive treatment and those who discontinued early. In this study with a limited population of elderly patients, a large difference between treatments would make the interpretation of the results difficult.

The inclusion of CCI data highlights the impact of comorbidities on treatment tolerability in older patients. Although this provides valuable insights, the lack of a Comprehensive Geriatric Assessment (CGA) is a limitation of our study. CGA offers a more nuanced evaluation of functional status, frailty, and overall fitness, which could further inform treatment decisions. Future prospective studies incorporating CGA would provide a more holistic understanding of the interplay between these factors and NAC outcomes in geriatric populations. The higher incidence of Grade 3–4 toxicities in older patients, particularly neutropenia, highlights the need for individualized management to maintain treatment tolerability. While G-CSF was used to manage toxicities, retrospective data limitations prevented detailed analysis of dose reductions, delays, or quality-of-life impacts. Prospective studies with standardized data collection are needed to better understand the toxicity spectrum and its effects on outcomes.

## Methods

### Study population and evaluation

This is a two-center retrospective cohort study approved by the Ethics Committee of Dokuz Eylul University (2023/08–11). Patients who underwent neoadjuvant treatment at two tertiary centers from 2010 to 2021 were retrospectively evaluated. A total of 535 patients who completed at least 80% of neoadjuvant chemotherapy were included in the study. This criterion was applied to minimize treatment heterogeneity and ensure that the results reflected the outcomes of patients who underwent most of their intended treatment. A total of 4 patients were excluded for not meeting this criterion, and baseline characteristics for these excluded patients were unavailable due to retrospective data limitations. Patients who did not undergo curative surgery for various reasons and patients with missing clinicopathologic data were excluded because the primary objectives of the study were to assess surgical outcomes, including pathologic complete response (pCR), breast-conserving surgery, and avoidance of lymph node dissection. While this selection criterion limits generalizability to the broader population receiving NAC, it ensures a focused evaluation of outcomes directly related to the surgical phase. Clinicopathologic and treatment-related characteristics were reviewed from patients’ files and electronic records of each tertiary cancer center, including age at diagnosis, menopausal status, tumor histology and differentiation, hormone receptor (HR) and HER2 expression status, Ki67 index, NAC regimen, pathologic response, and chemotherapy-related toxicities. The decision of NAC and surgical approach was determined by the institutional tumor board.

## Clinicopathological evaluation

Clinical and pathologic stages were assigned according to the 8th edition of the American Joint Committee on Cancer staging system^17^. Estrogen receptor (ER), progesterone receptor (PR), and HER2 status of pathology specimens were evaluated by accredited pathology laboratories at each center, no central evaluation was performed. Pathologic biomarker detection and reporting were performed according to the current ASCO-CAP guideline. ER and PR status was considered positive if staining was > 1% by IHC^18^. HER2 overexpression was defined as 3 + by IHC or 2 + by IHC and gene amplification by ISH^19^.

Patients were stratified into 5 different molecular subtypes according to the St Galen 2013 consensus. HER2-positive patients were evaluated in the same group with a treatment-oriented approach regardless of hormone status^20^.

Primary outcomes were T(tumor) and N(nodal) downstaging, pathologic response, and thype of breast and axillary surgery. Downstaging was defined as showing a pathologic stage lower than the clinical stage.

Neoadjuvant CT response was evaluated according to the MD Anderson Residual Cancer Burden Index by using an online calculator https://www3.mdanderson.org/app/medcalc/index.cfm? pageName=jsconvert3. pCR is defined as the absence of any invasive cancer in both breast and lymph nodes.

### Statistical analysis

The patients were divided into two groups based on age, with one group comprising patients aged 65 years and older and the other group comprising patients younger than 65 years. Pathologic response and surgical outcomes were compared between the two groups. The categorical variables were compared using Pearson’s chi-squared test or Fisher’s exact test. For descriptive analysis, percentages were used for categorical variables, and medians and ranges were used for continuous variables. The median follow-up period was evaluated by the reverse Kaplan-Meier method.

Unadjusted hazard ratios (HRs) for pCR and decisions regarding surgical subtypes were determined using logistic regression models. Variables included in the multivariate analyses were selected based on statistical significance in univariate analyses (*p* < 0.05). To account for potential confounding factors, adjusted HRs were calculated using multivariate regression analysis. Multicollinearity among the variables was assessed using the variance inflation factor (VIF), and all VIF values were below the cutoff of 5, indicating no significant multicollinearity.

All statistical analyses were performed with the SPSS Statistics 25.0 for iOS software program (SPSS, Inc., Chicago, IL, USA), and *P* < 0.05 was considered statistically significant.

## Conclusion

Older patients with breast cancer can derive similar benefits from NAC as their younger counterparts without significant differences in surgical and pathological outcomes. Age alone should not be a determining factor for the exclusion of patients from NAC. Further studies incorporating prospective designs and geriatric assessments are warranted to optimize treatment strategies for this population.

## Data Availability

All data are available upon reasonable request to the first author of the article.
